# Process–Structure–Properties in Polymer Additive Manufacturing

**DOI:** 10.3390/polym13071098

**Published:** 2021-03-30

**Authors:** Swee Leong Sing, Wai Yee Yeong

**Affiliations:** Singapore Centre for 3D Printing, School of Mechanical & Aerospace Engineering, Nanyang Technological University, 50 Nanyang Ave, Singapore 639798, Singapore; wyyeong@ntu.edu.sg

Additive manufacturing (AM) methods have grown and evolved rapidly in recent years. AM for polymers is an exciting field and has great potential in transformative and translational research in many fields, such as biomedical, aerospace, and even electronics. Current methods for polymer AM include material extrusion, material jetting, vat polymerisation, and powder bed fusion [1,2].

With the promise of more applications, detailed understanding of AM, from the processability of the feedstock to the relationship between the process-structure-properties of AM parts, have become more critical. More research work is needed in material development to widen the choice of materials for polymer additive manufacturing [3]. Modelling and simulations of the process will allow the prediction of microstructures and mechanical properties of the fabricated parts while complementing the understanding of the physical phenomena that occurs during the AM processes.

In this Special Issue, state-of-the-art review and current research, which focus on the process–structure–properties relationships in polymer additive manufacturing, are collated. In their review article, Dickson et al. looked into using fused filament fabrication, a type of material extrusion AM, for the fabrication of parts with fibre-reinforced thermoplastic composites [4]. For original research, Bere et al. looked into the manufacturing of complex parts with fibre-reinforced polymers using moulds fabricated by material extrusion AM [5]. Chen et al. looked into the crystallisation and thermal behaviours of poly(ethylene terephthalate)/biphenols complexes through melt post-polycondensation [6]. Abdolmaleki and Agarwala looked into using polyvinylidene difluoride added with barium titanate (PVDF–BaTiO_3_) for printed electronics [7]. Nagarajan et al. explored a methodology for the manufacture of magnetic composites using an in-house developed material jetting AM machine [8]. Sahay et al. demonstrated the use of a parallel plate for electrospinning-based AM in the fabrication of helicoidally arranged polyacrylonitrile fibre-reinforced polyvinyl alcohol polymer thin films [9]. Platek et al. analysed the deformation process of regular cell structures, manufactured using fused filament fabrication under quasi-static loading conditions [10]. Udroiu and Braga explored a new methodology for process capabilities analysis for material jetting AM [11]. Luis et al. studied the fabrication of silicone meniscus implants using a novel heat cured material extrusion technique [12]. Stoia et al. studied the mode I fracture toughness of polyamide and alumide samples fabricated using selective laser sintering [13]. Lastly, Andreaczyk et al. have developed a novel method for the experimental validation of numerically optimised turbomachinery components that are fabricated using AM [14].
